# ﻿New deep-sea species of *Aborjinia* (Nematoda, Leptosomatidae) from the North-Western Pacific: an integrative taxonomy and phylogeny

**DOI:** 10.3897/zookeys.1189.111825

**Published:** 2024-01-17

**Authors:** Julia K. Zograf, Alexander A. Semenchenko, Vladimir V. Mordukhovich

**Affiliations:** 1 A.V. Zhirmunsky National Scientific Center of Marine Biology, FEB RAS, 690041, Vladivostok, Russia A.V. Zhirmunsky National Scientific Center of Marine Biology Vladivostok Russia; 2 Federal Scientific Center of the East Asia Terrestrial Biodiversity, FEB RAS, 690022, Vladivostok, Russia Federal Scientific Center of the East Asia Terrestrial Biodiversity Vladivostok Russia; 3 Far Eastern Federal University, 690922, Vladivostok, Russia Far Eastern Federal University Vladivostok Russia

**Keywords:** DNA barcoding, free-living nematodes, Kuril-Kamchatka Trench, Leptosomatidae, phylogenetic relationships, scanning electron microscopy

## Abstract

Marimermithid nematodes parasitising invertebrates are mainly found in the deep-sea environments. Several adult and juvenile specimens marimermithids of the genus *Aborjinia* have been found in bottom sediments and inside Polychaeta during recent cruises to the Kuril-Kamchatka trench and the Kuril Basin (the Sea of Okhotsk). New species are described based on integrative study. *Aborjiniaprofunda***sp. nov.** differs from *A.eulagiscae* by the location of the ventral gland cell bodies (posterior to the nerve ring vs posterior to the cardia), by the smaller body size (23–28 mm vs 103–132 mm) and shorter tail (193–263 µm vs 500–850 µm). BI and ML phylogenetic analyses based on 18S and 28S rDNA suggest that genus *Aborjinia* belongs to the family Leptosomatidae. Based on molecular and morphological characters the new genus *Paraborjinia***gen. nov.** is proposed for *A.corallicola*. Within the family Leptosomatidae the new genus differs from all genera except *Aborjinia* by its endoparasitic lifestyle and hologonic ovaries. *Paraborjinia***gen. nov.** differs from *Aborjinia* by the position of cephalic sensitive organs (outer labial and cephalic papillae in two separate circles vs outer labial and cephalic papillae in one circle) and by the parasitic adult (vs free-living in *Aborjinia*).

## ﻿Introduction

In 1933 Ward described the very unusual nematodes *Thalassonemaophioctinis* Ward, 1933 from the brittle star *Ophioctenamitinum* Lyman, 1878 with an unclear taxonomical position. Forty years later Rubtzov and Platonova (1974) established a new family Marimermithidae which included two new genera *Trophomera* Rubtzov & Platonova, 1974 and *Marimermis* Rubtzov & Platonova, 1974. These nematodes resembled Mermithidae Braun, 1883 in lifestyle but differed in digestive system structure, anterior sensilla organization, and reproductive system. Later Rubtzov (1980) raised the family Marimermithidae to the rank of order. However, phylogenetic heterogeneity of the family was demonstrated in the following years. Simultaneously, the family Benthimermithidae Petter, 1980 also comprising internal parasites of marine invertebrates superficially similar to Mermithidae was described by Petter (1980). Later, Benthimermithidae was raised to the rank of order (Tchesunov 1995) and the genus *Trophomera* was transferred to the family Benthimermithidae, but the position of both orders (Marimermithida and Benthimermithida) in the nematode system remained uncertain. The rarity and peculiarities of the life cycle made studying these taxa difficult. Most species are known on the basis of only few, and often just a single, adult individual from sediments, so the host for most species remains unknown (Miljutin 2014a, b). Juveniles are found in the body cavities of a wide range of invertebrate hosts (e.g., Nematoda, Polychaeta, Priapulida, Bivalvia, Harpacticoida, Amphipoda, Isopoda); however, individuals without reproductive organs are usually unidentifiable (Miljutin 2014a, b). In addition, researchers often received samples fixed in formaldehyde, which limited the possibilities of investigation, particularly molecular studies.

Based on the morphological characters, Tchesunov (1997) suggested affinities between benthimermithids and Plectida and between marimermithids and Enoplia. Phylogenetic analyses based on a *Trophomera* SSU and LSU rDNA sequences provided support for a relationship between benthimermithids and plectids and resulted in the placement of all *Trophomera* sequences within the order Plectida (Tchesunov et al. 2009; Mardashova et al. 2011; Holovachov et al. 2013; Leduc and Zhao 2019). Leduc and Zhao (2019) proposed that the family Benthimermithidae be moved to the order Plectida.

The phylogenetic relationship of marimermithids remained unclear for many years. Marimermithid nematodes are parasites of invertebrates mainly found in deep-sea environments (Miljutin 2014a). Most marimermithids are large animals reaching several centimeters in length. Their morphology is quite simple and characterized by features usual for free-living nematodes, for example the presence of cephalic sense organs, a cylindrical pharynx, and a small cardium with a triradial internal lumen. On the other hand, their alimentary tract is often devoid of rectum and anus, caudal glands are absent or reduced in adults, hypodermal chords are hypertrophied, and the female genital system is adapted to facilitate the production of a large number of eggs. Such features are related to the parasitic way of life (Miljutin 2003).

The results from the phylogenetic analysis based on 18S rRNA of *Paraborjiniacorallicola* (Westerman et al., 2021) suggest a relationship with the family Leptosomatidae Filipjev, 1916 (Westerman et al. 2021). The recent phylogenetic analyses based on the sequences of genes 18S and 28S RNA of *Marimermismaritima* Rubtzov & Platonova, 1974 and *Aborjinia* sp. showed placement of these species within the order Enoplida but in the different branches of the tree (Tchesunov et al. 2022). Thus, molecular analyses supported the relationship of marimermithids with the Enoplida and did not justify the order Marimermithida as a holophyletic taxon.

Although the first representatives of *Aborjinia* Özdikmen, 2010 were described 40 years ago, they are rarely found, little-known, and poorly studied; only one species is known. Several adult and juvenile specimens of *Aborjinia* were found in bottom sediments and inside Polychaeta during recent cruises to the Kuril-Kamchatka trench and the Kuril Basin (the Sea of Okhotsk). Here we provide an integrative taxonomic study and determine the phylogenetic position of *Aborjinia*.

## ﻿Materials and methods

### ﻿Study sites and sampling

**Figure 1.**
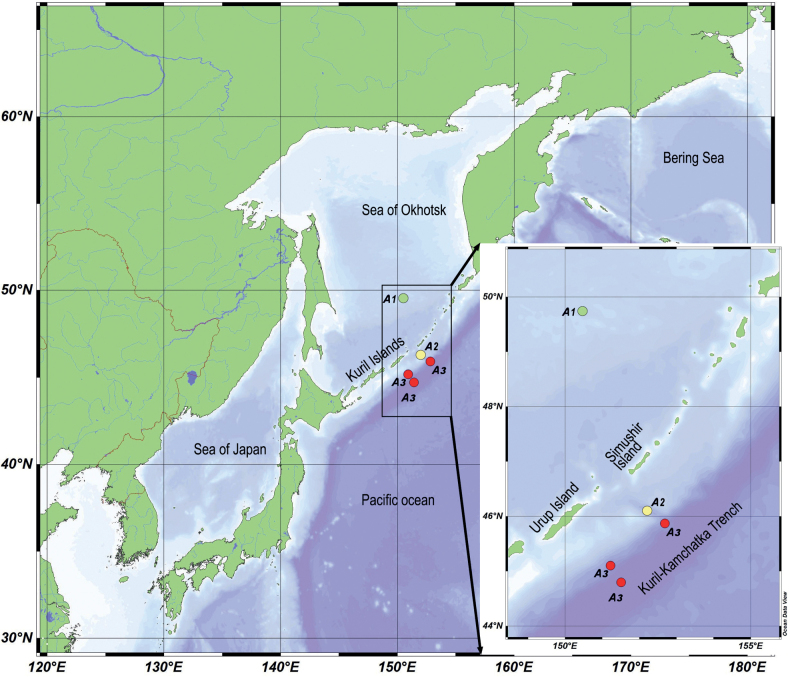
Map of the investigated area. Dots indicate sampled stations: A1 – *Aborjinia* sp. juvenile; A2 – *Aborjinia* sp. female; A3 – *Aborjiniaprofunda* sp. nov. male.

Specimens of *Aborjinia* were collected in several locations during KuramBio I (July-August 2012), SokhoBio (July-August 2015), and KuramBio II (August-September 2016) expeditions to the Kuril–Kamchatka Trench and adjacent northwest Pacific at water depths of 3350–9290 m (Fig. 1, Table 1). Samples collected by Agassiz trawl (AGT), epibenthic-sledge (EBS), and giant-box corer (GKG). On deck, the sediment from the AGT was sieved through a 1000-μm mesh size, and the upper layer of sediment (0–20 cm) from the GKG was carefully sieved through 1000-, 500-, and 300-μm mesh sizes. Immediately after sieving, samples from AGT and GKG were sorted in seawater using stereomicroscopes, and nematodes were removed and fixed in 4% buffered formaldehyde for morphological studies and in DESS (solution of 0.25 M disodium EDTA and 20% dimethyl sulfoxide (DMSO), saturated with NaCl, pH 8.0) for DNA studies. On deck, the samples from EBS were immediately transferred into chilled (-20 °C) 96% ethanol and stored in a -20 °C freezer for at least 48 h for subsequent DNA studies. In the laboratories of the ship and home institutes, sorting of the fauna was done on ice in order to avoid DNA decomposition.

**Table 1.** Localities, depth, and sampling data.

Species	Cruise	Gear	Area	GPS Coordinates	Depth [m]	Date [y/m/d]	Comments
*Aborjinia* sp. (specimen M11)	SokhoBio	Epibenthic sledge	6	48.0°N, 150.0°E	3347	2015/07/20	juvenile in body cavity of *Terebellides* sp.
*Aborjinia* sp. (specimen M10)	SokhoBio	Agassiz trawl	9	46.2°N, 152.1°E	3374	2015/07/25-27	female
*Aborjiniaprofunda* sp. nov.	KuramBio II	Agassiz trawl	A6	45.9°N, 152.8°E	6114	2016/08/25-27	male
*Aborjiniaprofunda* sp. nov.	KuramBio II	Box corer, Agassiz trawl	A9	44.7°N, 151.5°E	8235	2016/09/12-17	15 males
*Aborjiniaprofunda* sp. nov.	KuramBio II	Epibenthic sledge	A10	45.0°N, 151.1°E	5477	2016/09/16	male
*Leptosomatides* sp. (specimen L1, L2)	56 cruise of RV Akademic Oparin	Scuba-diver	49	56°25.405'N, 138°03.879'E	4-9	2019/08/02	female and juvenile

The NCBI database contains the *Leptosomatides* sequence HM564626, which has a very high level of similarity to the *Aborjinia* sequences. There is no unambiguous evidence in the literature of the incorrect identification of the indicated sequence, only an assumption (Tchesunov et al. 2022). Moreover, neither for the *Leptosomatides* TCR192 sample nor for *Aborjinia* sp. KKT is there any published morphological data. We were able to obtain two individuals (a female and a juvenile) of the genus *Leptosomatides* for morphological and molecular analysis and comparison with *Aborjinia*. Bottom sediments with specimens of *Leptosomatides* sp. were taken by scuba-divers in Ayan Bay (Sea of Okhotsk) in 2019 during a cruise onboard the R/V ‘Academic Oparin’ (Table 1). The sequences of these specimens are clustered within Leptosomatidae, and based on morphological analysis, specimens belong to genus *Leptosomatides* (Suppl. material 1). On deck, the sediment was sieved through 1000-, 500-, and 32-μm mesh sizes. Immediately after sieving, samples from 1000- and 500-μm mesh sizes were sorted in seawater using stereomicroscopes, and nematodes were removed and fixed in 10% formalin for morphological studies and in DESS for DNA studies. Half the samples from 32-μm mesh size were fixed with 4% buffered formaldehyde and half fixed with DESS.

### ﻿Morphological analysis

The male specimens of *Aborjinia* were picked out from the formaldehyde-fixed samples under a stereoscopic microscope, transferred to glycerin using the Seinhorst’s (1959) rapid method as modified by De Grisse (1969), and mounted on permanent slides. Drawings and DIC (differential interference contrast) photographs were made on an optical microscope Olympus BX 53 with the aid of a drawing tube and a digital camera, respectively.

The female specimen (DESS fixed, voucher M10) and a specimen of *Terebellides* sp. (Polychaeta: Terebellida: Trichobranchidae) with a parasitic juvenile of *Aborjinia* (ethanol fixed, voucher M11) in the body cavity were picked out from the fixative and placed in distilled water in a Petri dish. Photographs were made on an optical microscopes Nikon SMZ25 and Carl Zeiss Axio Observer 7 with the aid of a digital camera. After that, ~ 1 cm from the middle of the body of the nematode was cut off for genetic studies, and the remaining parts were placed back into the fixative.

Two male individuals fixed in formalin were cut to obtain pieces of the anterior end, middle part of pharynx, central part of body, and tail end. The specimens were then rinsed in the distilled water. After dehydration in graded ethanol series and ethanol-acetone mixture, the specimens were embedded in Spurr resin (Spur, Sigma). Semi-thin transverse sections (0.5 µm) were cut using a Leica Ultracat E Ultratome. The sections were first stained with methylene blue and azure II for 20 min at 60 °C and then with basic fuchsin for 4 min at room temperature (Humphrey and Pittman 1974), and mounted in Spurr resin on permanent glass slides. Photographs were made on an optical microscope Carl Zeiss AxioImager Z.2 with the aid of a digital camera. The acquired images were then adjusted for contrast and brightness using the ImageJ image processing software.

For the scanning electron microscopy, specimens were gradually dehydrated in a series of baths of increasing ethanol content, dried in a critical-point dryer, sputter-coated with gold, and observed and imaged with a Zeiss SIGMA 300VP scanning electron microscope (SEM).

The type material is deposited in the Senckenberg Museum, Frankfurt am Main, Germany (**SMF**) and in the Museum of the A.V. Zhirmunsky National Scientific Center of Marine Biology FEB RAS, Vladivostok, Russia (**MIMB**).

Abbreviations of the measured variables in the tables are as follows:

**a** body length divided by maximum body diameter;

**b** body length divided by pharyngeal length;

**c** body length divided by tail length;

**c**’ tail length divided by corresponding body diameter at cloacal level;

**L** body length (μm);

**V** distance of the vulva from the anterior end (μm);

**V (%)** distance of the vulva from the anterior end as percentage of body length (%).

### ﻿DNA extraction, sequence processing, phylogenetic inference, and secondary structure predictions

Nematodes were picked out from the DESS or ethanol fixed samples under a stereoscopic microscope. Specimens < 3 cm were mounted on temporary slides with sterile distilled water and observed at different magnifications using a light microscope (Olympus BX 53) with differential interference contrast, and equipped with a digital camera. Specimens > 3 cm were observed at different magnifications using a stereoscopic microscope Nikon SMZ25 equipped with a digital camera. After the vouchering DNA from the middle part of the body (~ 1 cm) was extracted using the Qiagen DNeasy extraction kit according to the protocol. PCR mixture contained 5 µl Go Taq Green Master Mix (Promega Corp., Madison, WI, USA), 0.5 µM of each primer, 3 μl of nuclease-free water (Ambion) and 1 µl of genomic DNA. Fragments of the nuclear ribosomal DNA and internal transcribed spacers (18S rDNA, ITS1, 5.8S rDNA, ITS2 and D2-D3 region of 28S rDNA) were amplified. For 18S rDNA, we used the primer set SSU_F_03 (f) and SSU_R_81 (r) (Blaxter et al. 1998) which amplifies a fragment of ~ 1800 bp. We used additional primers to sequence 18S rDNA amplicons: SSU_F_24_1 (f) (Meldal et al. 2007) and MN18R (r) (Floyd et al. 2005). The D2-D3 region of the 28S ribosomal DNA region was amplified using the primers D2a (f) and D3b (r) (Nunn 1992). The length of the obtained amplicon was ~ 700 bp. The internal transcribed spacer (ITS, includes ITS1, 5.8S rDNA and ITS2) region was amplified with the primers Vrain2F and Vrain2R (Vrain et al. 1992) which amplifies a fragment of ~ 1200 bp. The length of the obtained amplicon was 700 bp. PCR products were visualized on a 1.5%-TBE agarose gel GelDoc XR+ imaging systems (BioRad). Each PCR fragment was purified using Exonuclease I (ExoI) and Thermosensitive Alkaline Phosphatase (FastAP) (Thermo Fisher Scientific Inc., USA). PCR products were bidirectionally cycle sequenced using BigDye Terminator v. 3.1 Cycle Sequencing Kit (Applied Biosystems, Inc.), and bidirectionally sequenced on an ABI 3130XL automated sequencer using BigDye Terminator v. 3.1 Cycle Sequencing Kit (Applied Biosystems, Inc.). Sequences were manually assembled and edited using Finch TV and MEGA 7 (Kumar et al. 2016). Also, MEGA7 was used for calculated inter- and intraspecific p-distances.

The 18S and 28S rDNA sequences were checked and aligned at the nucleotide level using T-Coffee algorithm (Magis et al. 2014) on a MPI Bioinformatics Toolkit web service (Zimmermann et al. 2018). Bayesian phylogenetic analyses were conducted with MrBayes v. 3.2.7a (Ronquist et al. 2012). For tree reconstruction, we used the obtained sequences, as well as dataset from GenBank belonging to family Leptosomatidae, with lengths longer than 1000 bp for 18S and 650 bp for D2-D3 region 28S rDNA. PartitionFinder 2.1.1 (Lanfear et al. 2012) was used to select the best-fit partitioning scheme and models for each loci using the greedy algorithm with linked branch lengths for the corrected Bayesian Information Criterion as the optimality criterion for model selection. The best models for both ribosomal loci were SYM+I+G. Bayesian Inference was performed with two independent runs of Metropolis-coupled Markov chain Monte Carlo analyses, with each run comprising one cold chain and three heated chains at a default temperature setting of 0.1. The chains were run for 10 million generations and sampled every 100 generations. A burn-in of 2.5 million generations (or 25% of the sampled trees) was used. Moreover, trace files were visually inspected in Tracer 1.7 (Rambaut et al. 2018). We conducted Maximal likelihood (ML) analyses in IQ-Tree v. 2.2.0 (Minh et al. 2020) with 1 million ultra-fast bootstrap replications (Hoang et al. 2018) with model finding (Kalyaanamoorthy et al. 2017) algorithms. FigTree v. 1.4.4 was used to visualize phylogenetic trees after analysis.

ITS2 boundaries were identified by using hidden Markov models implemented in the ITS2 Ribosomal RNA Database (http://its2.bioapps.biozentrum.uni-wuerzburg.de/; Ankenbrand et al. 2015). The common folding pattern of ITS2 molecules for *Aborjinia* spp. was found by running the multilign and TurboFold algorithms on the RNAstructure webserver (http://rna.urmc.rochester.edu/RNAstructureWeb; Reuter and Mathews 2010) using the default parameters. We used 4SALE (Seibel et al. 2008) to generate the consensus secondary structure of our dataset after alignment sequence structures in ITS2 Ribosomal RNA Database. CBCAnalyzer (Wolf et al. 2005) was used to detect CBCs and hemi-CBCs (one-sided substitutions before CBCs) from aligned matrix.

## ﻿Results

### ﻿Taxonomic account

#### ﻿Family Leptosomatidae

Taxon classificationAnimaliaEnoplidaLeptosomatidae

Filipjev, 1916

8D70876E-03CE-5E76-829E-FE653753A455

##### Diagnosis

**(Smol et al. 2014; emended).** Large nematodes (up to 172 mm). Six inner labial sensilla mostly papilliform, six outer labial and four cephalic sensilla papilliform or setiform. Amphids pocket-shaped. Large number of metanemes with caudal filament usually present: dorsolateral and ventrolateral or only dorso-lateral orthometanemes and loxometanemes of type I. Many species with ocelli. Buccal cavity narrow, sometimes with tooth-like thickening. Pharynx inserts into the body cuticle in the region of buccal cavity, the cephalic capsule is variable in the form. Three pharyngeal glands open in the buccal cavity. Pharynx always smooth in outline. Secretory-excretory system, if present, usually restricted to the pharyngeal region, may consists of two cells. Female reproductive system didelphic-amphidelphic with antidromously reflexed ovaries. Males with two testes opposed. Gonad positions relative to intestine variable in species, with anterior and posterior gonad position reversed. Subventral or ventral precloacal papillae (never tubules) often present. Caudal glands mostly present, extending into the precaudal region. Marine and parasites of marine invertebrates.

#### ﻿ Aborjinia

Taxon classificationAnimaliaEnoplidaLeptosomatidae

Özdikmen, 2010

CB4B3E0E-102D-5F5E-A9BB-4EC4E61C5B43

##### Diagnosis

**(emended after Tchesunov and Spiridonov 1985; Miljutin 2003, 2014a).** Very large nematodes; at the larval stage parasitize marine invertebrates. Adult worms are free-living. Three lips. Pharynx cylindrical, muscular, with tri-radial internal lumen. Rectum and anus present. Outer labial and cephalic sensilla papilliform, situated in one circle. Amphideal fovea small, pore-like. Cervical setae absent. Excretory-secretory system consists of two cells. Female reproductive system didelphic, amphidelphic, ovaries hologonic. Male reproductive system didelphic, testes outstretched. Tail convex-conoid, broadly rounded.

##### Type species.

*Aborjiniaeulagiscae* (Tchesunov & Spiridonov, 1985): Özdikmen 2010, by original designation [= *Australonemaeulagiscae* Tchesunov & Spiridonov, 1985].

##### Invalid species.

*Aborjiniacorallicola* Westerman, de Moura Neves, Ahmed & Holovachov, 2021.

= *Paraborjiniacorallicola* (Westerman, de Moura Neves, Ahmed & Holovachov, 2021), comb. nov.

#### ﻿ Aborjinia profunda sp. nov.

Taxon classificationAnimaliaEnoplidaLeptosomatidae

D3262D83-144D-5A08-BD6D-6FDC4E1E1D0E

https://zoobank.org/FA1FD587-B2E2-49BB-996B-57F2D15AD74E

**Figure 2.**
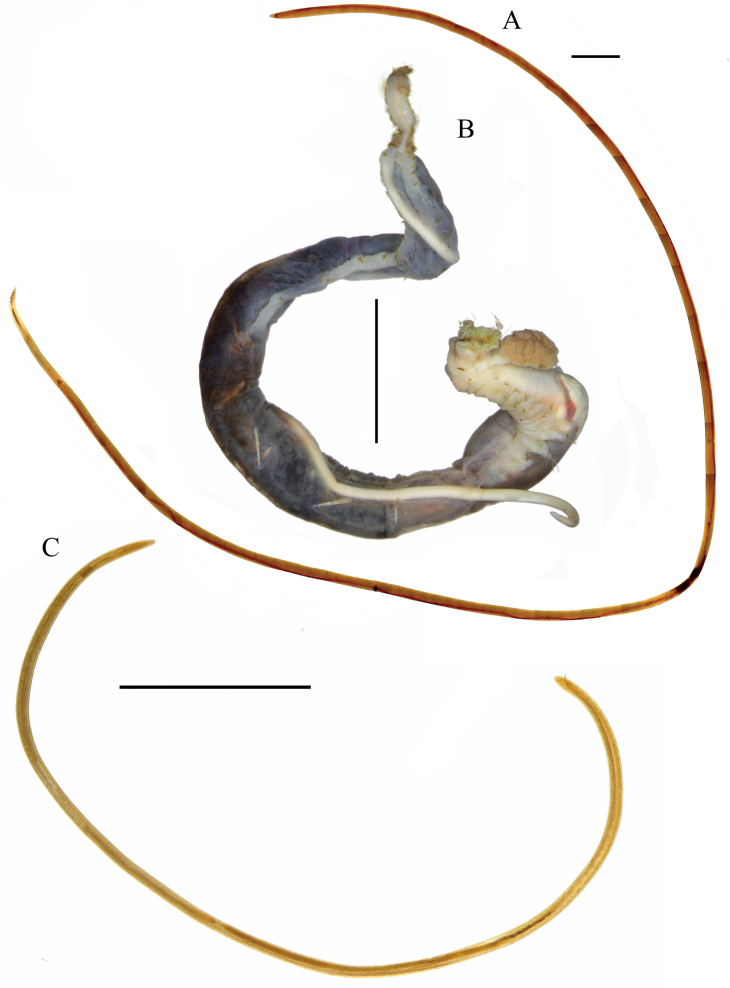
Examined species of *Aborjinia*, entire bodies, light microscopy **A***Aborjinia* sp., female (assembled panorama) **B***Terebelus* sp. with juvenile of *Aborjinia* sp. **C***Aborjiniaprofunda* sp. nov., male. Scale bars: 5000 µm.

**Figure 3.**
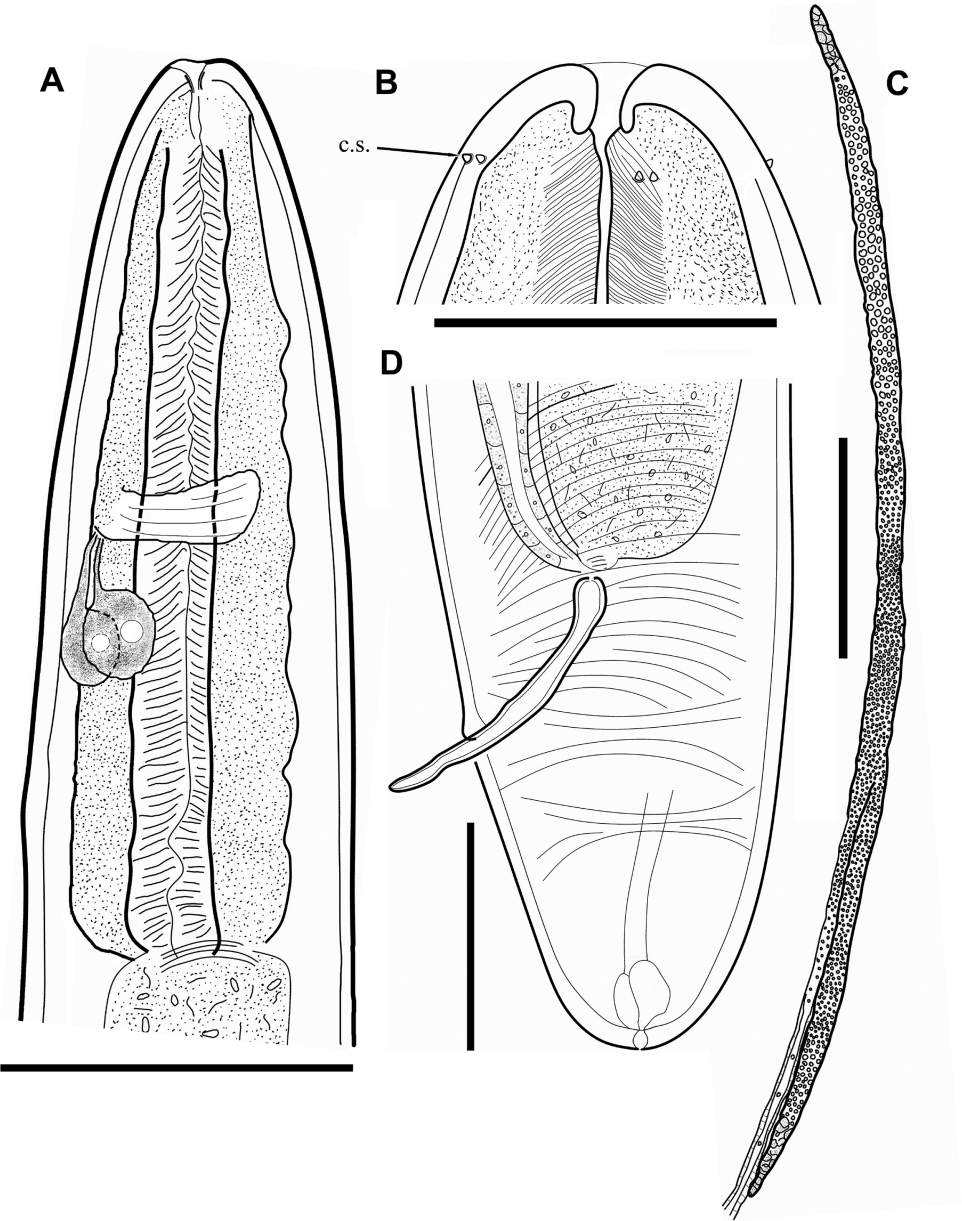
*Aborjiniaprofunda* sp. nov. **A** anterior end of male **B** head of male **C** male reproductive system **D** male tail. Abbreviation: c.s – cephalic sensilla. Scale bars: 100 µm (**B**); 200 µm (**D**); 500 µm (**A**); 2000 µm (**C**).

**Figure 4.**
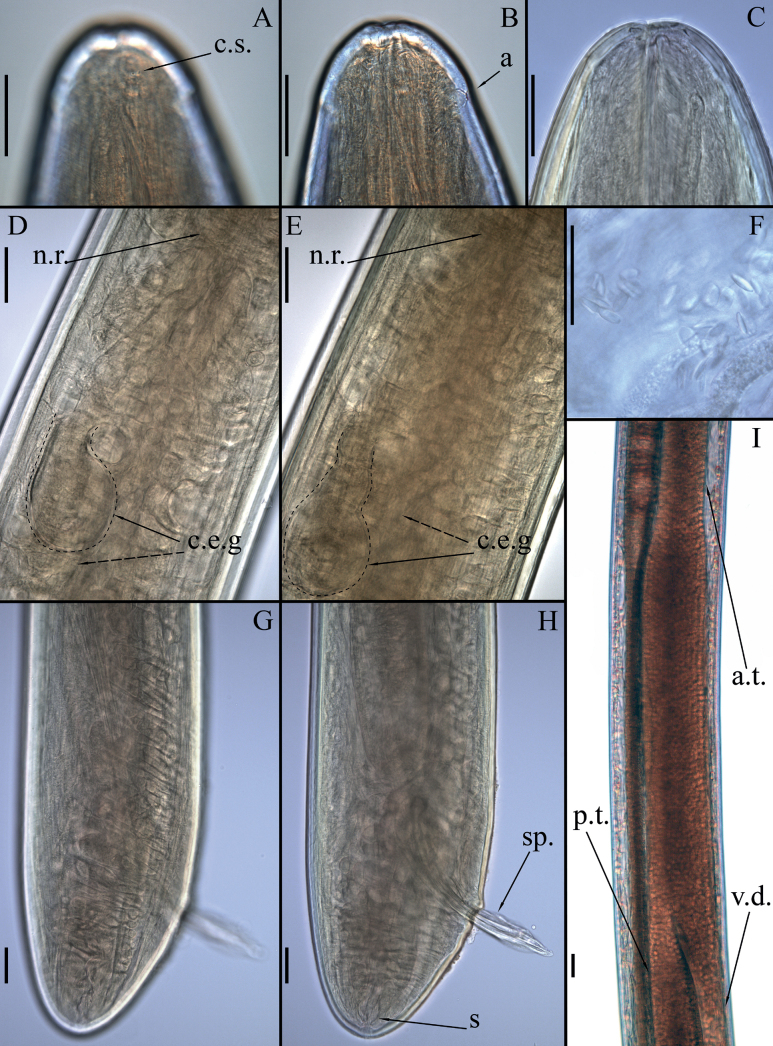
*Aborjiniaprofunda* sp. nov., male. Light microscopy, DIC **A–C** head **D, E** cellular bodies of the cervical excretory gland **F** crystalloid bodies **G, H** posterior end region **I** the vesicula seminalis region. Abbreviations: a – amphid, a.t. – anterior testis, c.s. – cephalic sensillum, c.e.g. – cervical excretory gland, n.r. – nerve ring, p.t. – posterior testis, s. – spinneret, sp. – spicules, vd – vas deferens. Scale bars: 50 µm.

**Figure 5.**
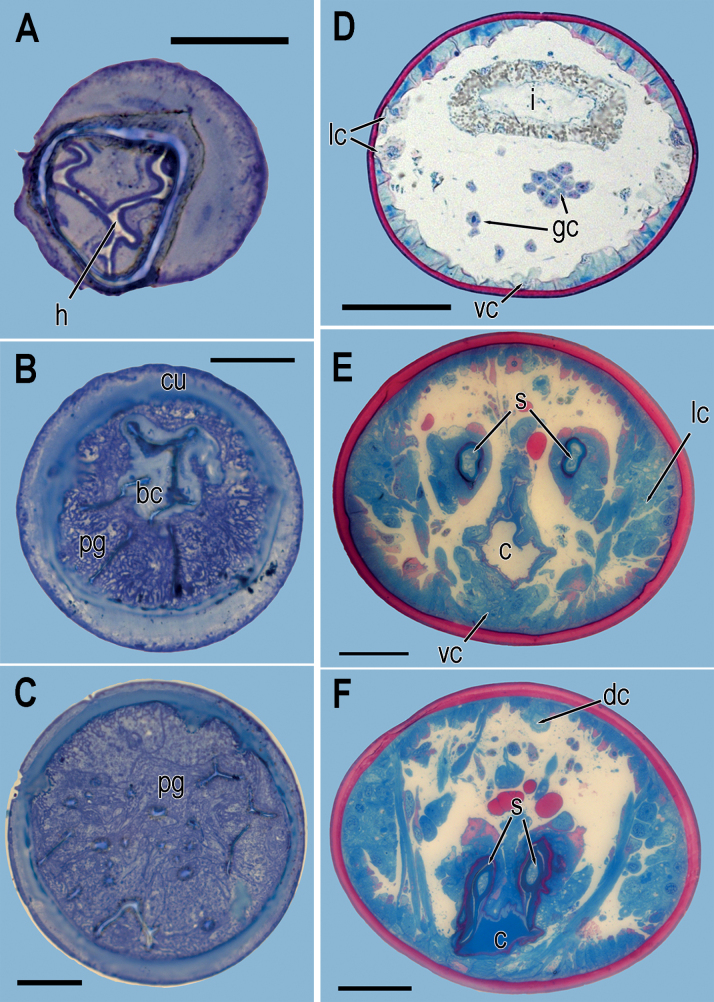
*Aborjiniaprofunda* sp. nov. Light microphotographs of transverse sections **A** buccal cavity at the upper level of the head (h) **B** buccal cavity surrounded with pharyngeal glands **C** pharyngeal region tightly filled with pharyngeal glands bodies **D** midbody with intestine and gonad **E** posterior region at the level of distal part of spicules **F** posterior region close to cloacal opening. Abbreviation: bc – buccal cavity, c – cloaca, cu – cuticle, gc – germinal cells, h – heilostoma, i – intestine, lc – lateral chords, pg – pharyngeal glands, s – spicules, vc – ventral chords. Scale bars: 20 µm (**A–C**); 50 µm (**E, F**); 100 µm (**D**).

**Figure 6.**
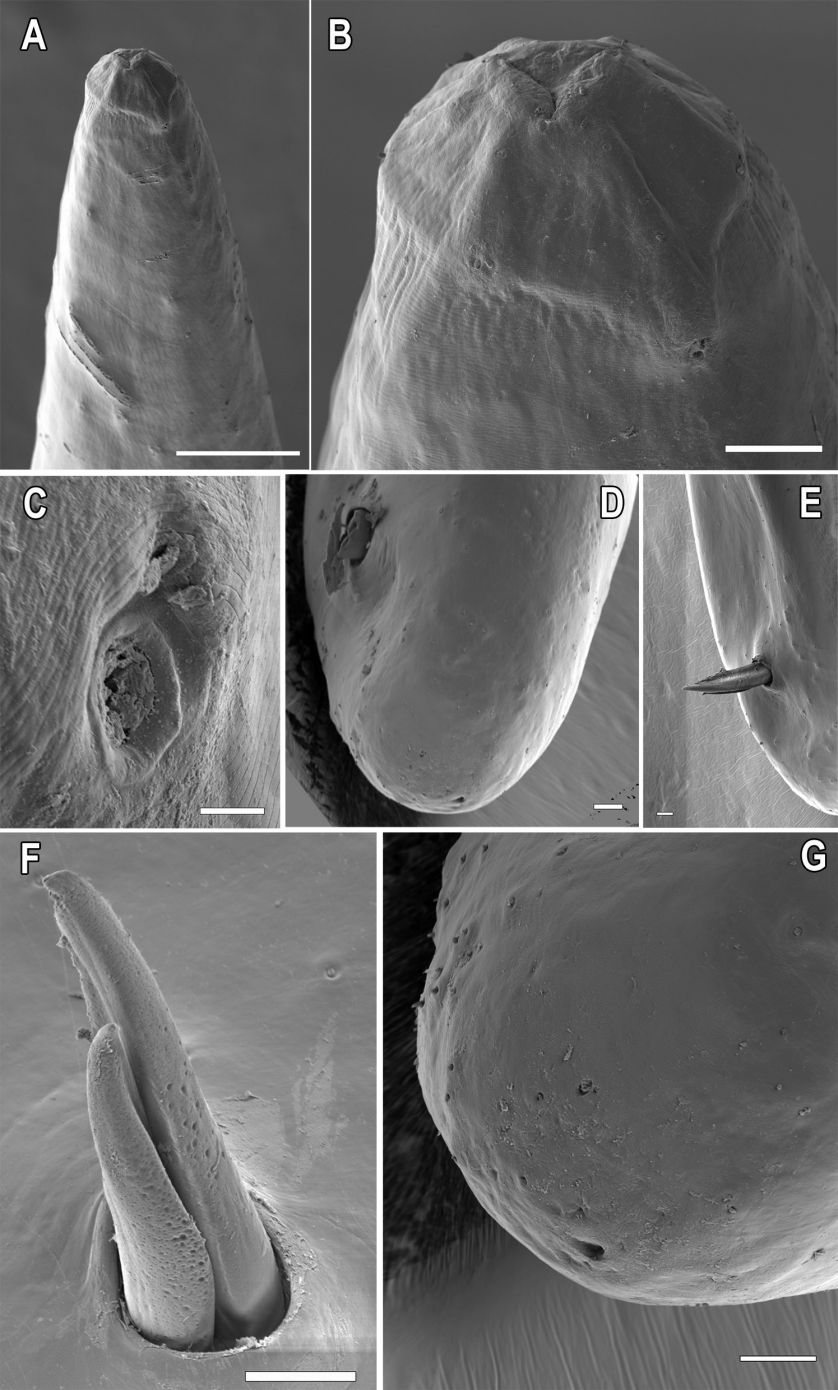
*Aborjiniaprofunda* sp. nov. SEM**A** anterior end of male **B** head of the male **C** amphideal fovea **D** posterior end of the male **E** spicules protruding from cloacal opening **F** spicules **G** tail tip with spinneret opening. Scale bars: 2 µm (**C**); 20 µm (**B, D, E, F, G**); 100 µm (**A**).

Figs 2 , 3 , 4 , 5 , 6

##### Diagnosis.

Body 22.9–27.7 mm long in males. Six outer labial and cephalic sensilla papilliform, situated 19–21 µm from anterior end. Amphideal aperture located 37–40 µm from anterior end. Pharynx tubular without any valves or bulbs, tightly surrounded by the glandular tissue. Nerve ring situated ~ 40% of pharynx length from anterior end. Intestine well developed with wide lumen. Spicules slightly bent, 364–372 µm long. No pre- or postcloacal sensilla or supplements. Spinneret present.

##### Type material examined.

Three males (holotype and two paratypes). The holotype (SMF 14457) and paratype (SMF 14458) are deposited in the Senckenberg Museum, Frankfurt am Main, Germany. Paratype (MIMB 42307) is deposited in the Zoological Museum of A.V. Zhirmunsky National Scientific Center of Marine Biology, Vladivostok, Russia.

##### Other material examined.

One formalin-preserved specimen (male) and two DESS-preserved specimens (males). The Kuril-Kamchatka Trench, water depth 5477 m (45.0°N, 151.1°E), 6114 m depth (45.9°N, 152.8°E) Deposited in the Zoological Museum of A.V. Zhirmunsky National Scientific Center of Marine Biology, Vladivostok, Russia (MIMB 42308).

##### Type locality.

The Kuril-Kamchatka Trench, water depth 8235 m (44.7°N, 151.5°E) (Fig. 1, Table 1).

##### Additional locality.

The Kuril-Kamchatka Trench, water depth 5477 m (45.0°N, 151.1°E), 6114 m depth (45.9°N, 152.8°E) (Fig. 1, Table 1).

##### Etymology.

Species name derived from the Latin *profundus* that means deepwater and refers to the deepwater habitat of described species.

##### Nucleotide sequences.

GenBank accession numbers OP600452.1, OP600453.1 (small subunit ribosomal RNA gene, partial sequence; internal transcribed spacer 1, 5.8S ribosomal RNA gene, and internal transcribed spacer 2, complete sequence; and large subunit ribosomal RNA gene, partial sequence); OP407645.1, OP407646.1 (large subunit ribosomal RNA gene, partial sequence).

##### Description.

Large nematodes, 22.9–27.7 mm long, with an average diameter 0.2–0.4 mm. Body cylindrical, tapering towards both extremities (Figs 2, 3). The cuticle finely striated under SEM, and thin (~ 5 µm; Fig. 6). Hypodermis and muscle layers are thin, cords are prominent (Fig. 5). Body pores distinct, irregularly arranged. Measurements tabulated in Table 2.

**Table 2.** Morphometrics (μm) of *Aborjiniaprofunda* sp. nov. and *Aborjinia* sp. (abbreviations of characters defined in the Materials and methods).

	*Aborjiniaprofunda* sp. nov.			*Aborjinia* sp.
	HT♂	♂	♂	HT ♀
L	27740	22900	25700	172000
Tail length	263	218	193	524
Nerve ring from anterior end	500	513	501	
Head diam. at level of cephalic setae	91	83	87	
Anal body diam.	289	217	230	467
Maximum body diam.	355	264	290	850
Pharyngeal length	1250	1199	1158	2148
Amphid from anterior end	33	37	43	
Spicule length	364	297	335	
Renetta cells from anterior end	792	742	725	5053
a	78.1	86.7	88.6	202.3
b	22.2	19.1	22.2	78.8
c	105.5	105	133.2	328.2
c’	0.91	1	0.83	1.1

***Head*** narrow, bluntly rounded with three lips. Inner labial sensilla papilliform, hardly visible under light microscope. Papilliform outer labial sensilla and cephalic sensilla in one circle, 1–2 µm long, situated 19–21 µm from anterior end (Fig. 6A, B). Amphideal opening pore-like, located 37–40 µm from anterior end. Pharynx tubular without any valves or bulbs, tightly surrounded by the glandular tissue (Figs 3A, 4). Nerve ring situated ~ 40% of pharynx length from anterior end. Intestine well developed with wide lumen. Ventral gland consists of two cells. Cell bodies ~ 80 μm long and 75 μm wide (~ 30% of corresponding body diam.), arranged in tandem and situated 725–792 µm from anterior end (Fig. 4D, E). Excretory pore not observed.

***Male reproductive system*** didelphic, testes outstretched. Spicules paired, symmetrical, slightly bent, 364–372 µm long (Figs 3, 4). Gubernaculum not found. No pre- or postcloacal sensilla or supplements. Rectum and anal opening functional. Tail conical with terminal spinneret, caudal glands not observed.

##### Remarks.

The new species differs from *A.eulagiscae* by the location of the ventral gland cell bodies (posterior to the nerve ring vs posterior to the cardia), by the smaller body size (23–28 mm vs 103–132 mm), and the shorter tail (193–263 µm vs 500–850 µm).

#### ﻿ Aborjinia

Taxon classificationAnimaliaEnoplidaLeptosomatidae

sp.

BCCF9A45-DFA2-5D5D-AFC3-128C6A22AD91

Figs 7 , 8

##### Material examined.

Anterior and posterior parts of the DESS fixed female. Eastern slope of the Kuril Islands, water depth 3374 m (Fig. 1, Table 1).

##### GenBank accession numbers.

OP600454.1 (small subunit ribosomal RNA gene, partial sequence; internal transcribed spacer 1, 5.8S ribosomal RNA gene, and internal transcribed spacer 2, complete sequence; and large subunit ribosomal RNA gene, partial sequence); OP407647.1 (large subunit ribosomal RNA gene, partial sequence).

##### Description.

**Figure 7.**
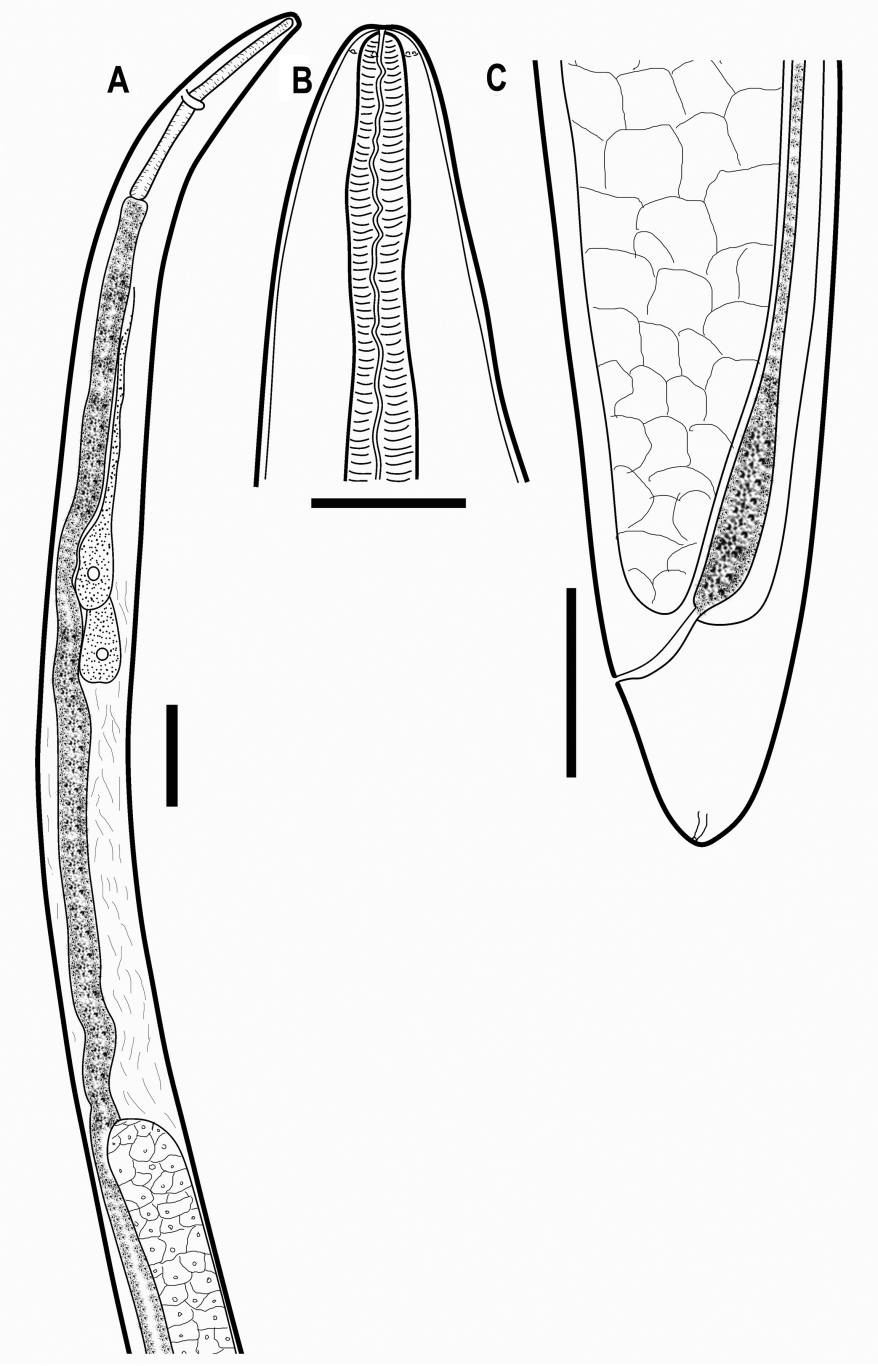
*Aborjinia* sp. **A** anterior end of female **B** head end of female **C** tail of female. Scale bars: 250 µm (**B**); 500 µm (**C**); 1000 µm (**A**).

**Figure 8.**
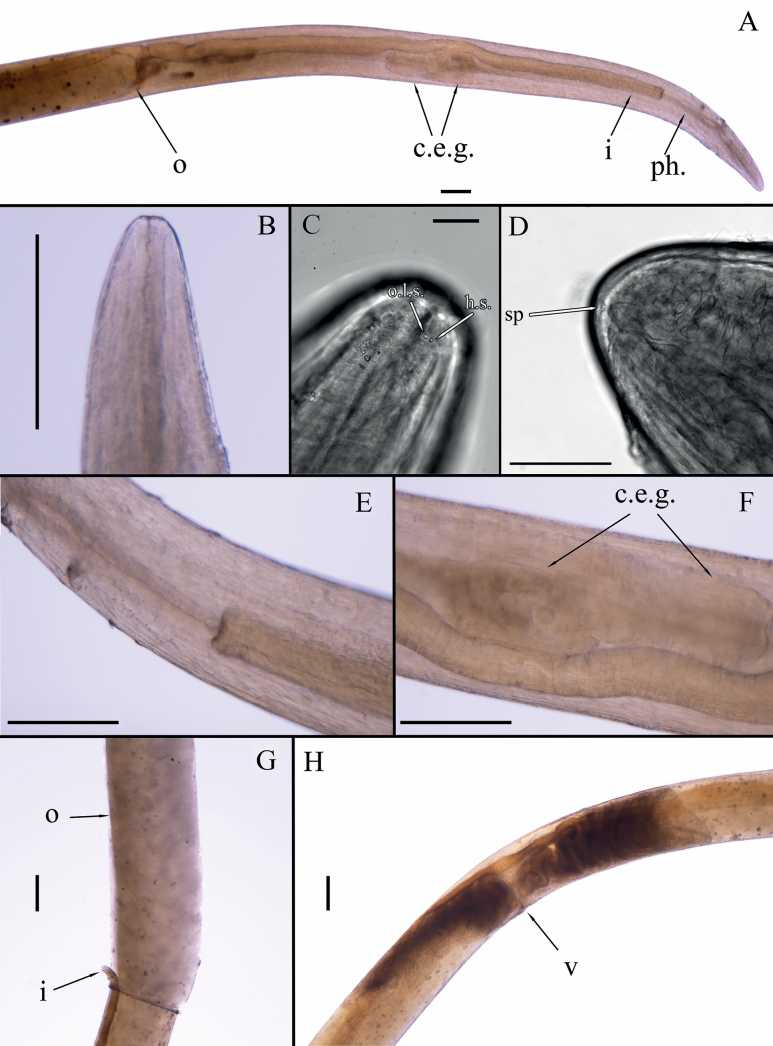
Light microscopy, DIC. *Aborjinia* sp., female **A** anterior end **B** head **C** anterior end with cephalic sensilla **D** tail end with spinneret **E** pharynx-intestine connection **F** cellular bodies of the cervical excretory gland **G** ovary **H** vulva region. Abbreviation: c.e.g. – cervical excretory gland, h.s. – cephalic sensilla; i – intestine, o – ovary, o.l.s. – outer labial sensilla, ph – pharynx, v – vulva. Scale bars: 50 μm (**C**); 100 μm (**D**); 500 µm (**A, B, E, F–H**).

Body opaque, cylindrical, slightly narrowing to both ends, 17.2 cm long (Figs 2, 7). Cuticle smooth under the light microscope. Inner labial sensilla papilliform, hardly visible under light microscope. Papilliform outer labial sensilla and cephalic sensilla in one circle (Fig. 8C). Amphideal opening pore-like. Buccal cavity small, narrow. Pharynx tubular without any valves or bulbs, cardia small. Intestinal lumen distinct only in its anterior most part. Secretory-excretory system consists of two big cells situated 5053 µm from anterior end (Fig. 8F). Excretory pore not observed. Reproductive system didelphic, amphidelphic with outstretched ovaries. Uteri large, tubular. Vulva located at midbody, a transverse slit. Tail conico-cylindrical. Anal opening present. Spinneret very vestigial (Fig. 8D), caudal glands not observed.

##### Molecular analysis.

In total, six specimens were sequenced for the 18S rDNA, 28S rDNA (D2-D3 region), and ITS (including ITS1, 5.8S rDNA, and ITS2) genes. Of these, two specimens belong to *Leptosomatides* Filipjev, 1918, two specimens to *Aborjiniaprofunda* sp. nov., and one female and one juvenile belong to *Aborjinia* sp. The sequence length of 18S rDNA was 1694–1716 bp (14 variable sites, 11 parsimony-informative characters), 28S rDNA – 659–671 bp (66 variable sites, 59 parsimony-informative characters). The length of ITS for the genus *Aborjinia* was 1054–1094 bp (61 variable sites, 9 parsimony-informative characters) whereas for *Leptosomatides* sp. length was 1267 bp (3 variable sites).

To calculate genetic distances, as well as to reconstruct phylogenetic relationships, we used all available sequences of leptosomatids from GenBank (Suppl. materials 2, 3). The BI phylogeny using 18S rRNA reveal *Deontostoma* Filipjev, 1916 as the earliest branching lineage within Leptosomatidae. However, this genus was shown to be polyphyletic which occupied two of the branches of the polytomous clade. A well supported clade (Bayesian PP, BPP = 1; ML bootstrap value percent, ML = 89) united the four samples of *Pseudocella* Filipjev, 1927. Another moderately supported clade uniting *Thoracostomamicrolobatum* and two species of genus *Proplatycoma* Platonova, 1976 was uncovered (BPP = 0.78). Two species of *Platonova*Mordukhovich et al., 2019 and *Synonchus* Cobb, 1894 were placed as monophyletic clade with moderate support (BPP = 0.96; ML = 98), whereas the placement of *Cylicolaimus* de Man, 1889 was unsupported. *Thoracostomatrachygaster* Hope, 1967 was sister to obtained sequences and *Paraborjiniacorallicola* (BPP = 0.99, ML = 86) and not monophyletic to *Thoracostomamicrolobatum* and *Thoracostoma* sp., rendering that genus polyphyletic. A clade containing two samples of *Leptosomatides* (vouchers L1, L2) and a clade uniting samples of *Aborjinia* (excluding *Paraborjiniacorallicola*) and *Leptosomatides* (HM564626) were high supported (BPP = 1; ML = 94 and BPP = 0.99; ML = 96, respectively). *Paraborjiniacorallicola* was sister to previous clade with high Bayesian support (BPP = 0.99, ML = 72).

The phylogenetic relationships using 28S rRNA reveals opposite topology compared with 18S rRNA. *Paraborjiniacorallicola*, the rest *Aborjinia* species and *Leptosomatides* (vouchers L1, L2) were the earliest branching lineages but supports of these clades were moderate or low. Genus *Thoracostoma* was also polyphyletic. *Deontostoma* was placed in one clade with *Thoracostomamicrolobatum* (BPP = 0.99, ML = 83). *Pseudocella* and one out of three *Thoracostoma* were sister to *Platonova* (including *Synonchus*) (BPP = 0.95, ML = 88).

The average intergeneric p-distances within Leptosomatidae were 1.96% (0.57%–4.66%) and 13.30% (9.15%–17.12%) for 18S rDNA and 28S rDNA respectively if the two non-monophyletic species *Thoracostomatrachygaster* and *Thoracostomamicrolobatum* Allgén, 1947 as well as *Paraborjiniacorallicola* and the remaining *Aborjinia* belongs to different genera. Genus *Aborjinia* (including sequences HM564626 and HM564855, excluding *Paraborjiniacorallicola*) differed from other genera of the family by 1.49% and 11.57% in average for 18S rDNA and 28S rDNA, respectively. Same values for genus *Leptosomatides* were 1.71% and 13.84% and for *Paraborjiniacorallicola* were 3.78% and 16.15%, respectively.

**Figure 9.**
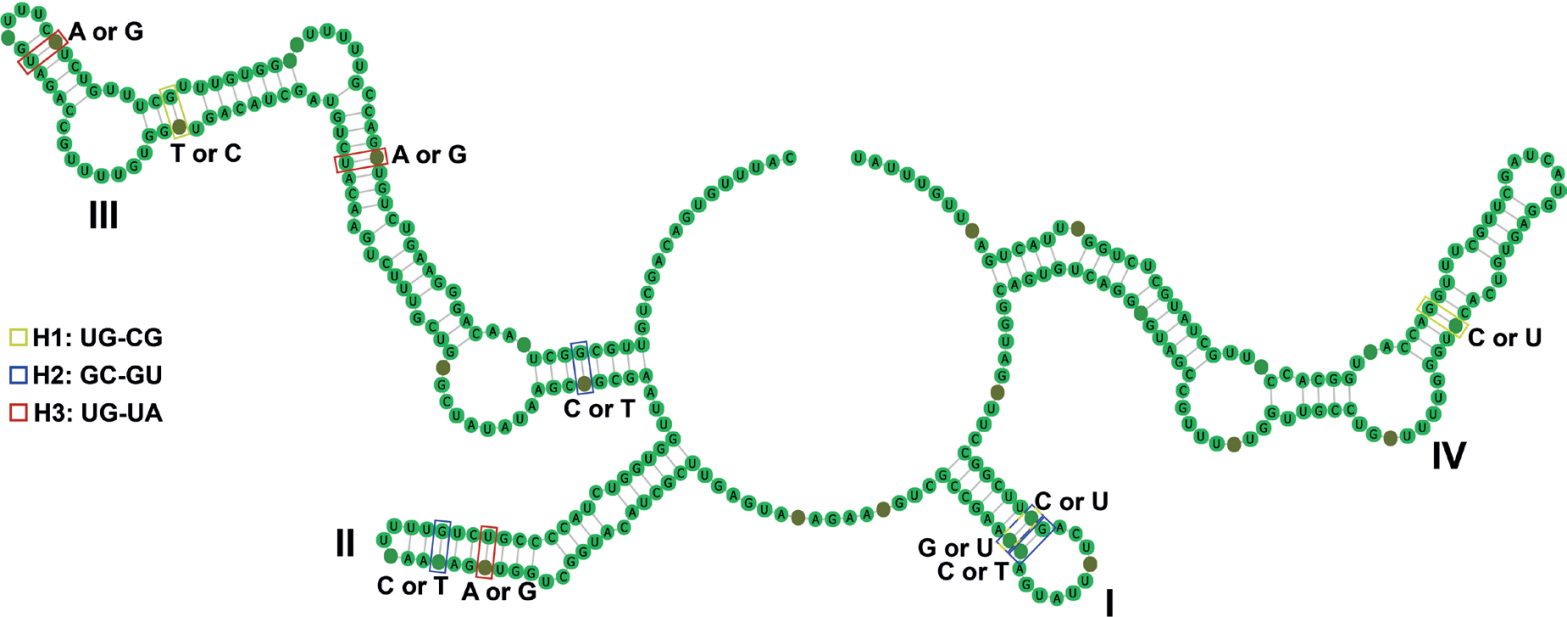
Consensus ITS2 secondary structure and the hemi compensatory base change (hemi-CBCs) derived from *Aborjinia* species. The four stems are labelled. Each type of base change (H1-H3) indicates by unique color. CU and GU change that relates to H1 or H2 depending on comparison pair.

The interspecific p-distance for 18S rDNA between *Aborjiniaprofunda* sp. nov. and *Aborjinia* sp. (voucher M10) was 0.24%, for 28S rDNA this value was 1.82%, and for ITS2 5.78% (Table 3). Using the programs RNA structure and 4SALE, homologous regions of *Aborjinia*ITS2 as well as *Aborjinia* sp. MZ504143 sequences were generally folded as comparable secondary structural motifs. Analyses revealed single secondary structure for all sequences contained four universal helices (Fig. 9). Comparison of sequences across taxa identified several hemi-compensatory base changes (hemi- CBCs, Table 4, Fig. 9) which in turn belonged to different types of changes (H1-H3). Various comparison pairs of *Aborjinia* species gave 3–7 hemi-CBCs while no double-sided changes (CBC) were found (Table 4).

**Table 3.** Interspecific p-distances (%) between the obtained sequences. Distances for ITS and 28S are above and below the diagonal, respectively (“-“ – data absent).

Taxon	1	2	3	4	5	6	7
1. *Aborjiniacorallicola*		–	–	–	–	–	–
2. *Aborjinia* sp. (specimen М10)	15.85		5.78	4.62	4.34	–	24.11
3. *Aborjiniaprofunda* sp. nov.	16.46	1.82		3.47	4.34		23.21
4. *Aborjinia* sp. (specimen М11)	15.85	1.37	1.37		3.18		24.11
5. *Aborjinia* sp. (MZ504143)	16.02	1.22	1.83	1.22		–	24.11
6. *Leptosomatides* sp. (HM564855)	15.87	1.25	1.88	1.72	1.57		–
7. *Leptosomatides* sp. (specimen L1, L2)	16.07	9.02	8.72	8.72	9.04	9.15	

**Table 4.** Number of CBCs (above diagonal) and hemi-CBCs (under diagonal) in the ITS2 secondary structure.

Taxon	*Aborjinia* sp. (specimen М10)	*Aborjiniaprofunda* sp. nov.	*Aborjinia* sp. (specimen М11)	*Aborjinia*_sp. (MZ504143.1)
*Aborjinia* sp. (specimen М10)		0	0	0
*Aborjiniaprofunda* sp. nov.	6		0	0
*Aborjinia* sp. (specimen М11)	5	3		0
*Aborjinia* sp. (MZ504143)	5	7	4	

##### Remarks.

**Figure 10.**
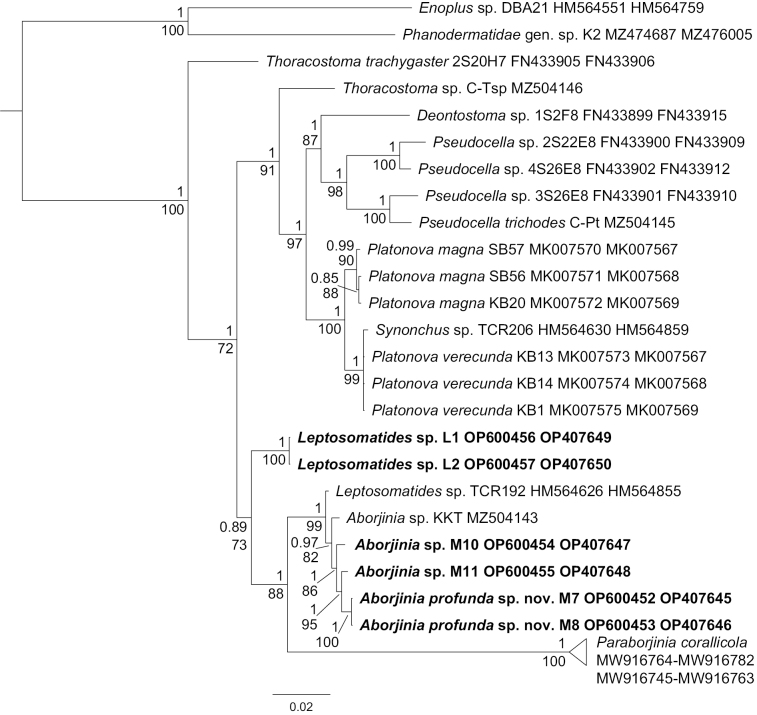
Bayesian phylogeny of the family Leptosomatidae, using concatenated 18S and 28S rDNA and SYM+ G model of nucleotide substitution. *Enoplus* sp. (Enoplidae) and Phanodermatidae gen. sp. were used as outgroup to root tree. Bayesian posterior probabilities (PP) are given above tree nodes and bootstrap support values found in the ML analysis are shown below nodes. Specimens obtained in this study are in bold.

To date only three species (including the present material) were originally described in the genus *Aborjinia*: *Aborjiniacorallicola*, *Aborjiniaeulagiscae*, and *Aborjiniaprofunda* sp. nov. but *Aborjiniacorallicola* is here transferred to *Paraborjinia* sp. nov. Both species (*A.eulagiscae* and *A.profunda* sp. nov.) are characterized by the outer labial and cephalic sensilla situated in one circle and the presence of two cells of secretory-excretory system. In the description of *P.corallicola* provided by Westerman et al. (2021) it is mentioned that outer labial sensillae and cephalic sensilla are situated in one circle. However, on the photograph provided in that paper these sensilla are situated in two separate circles. In addition, in *P.corallicola* the secretory-excretory system was not found, contrasting with the two giant and clearly visible cells in other *Aborjinia*. It should also be noted that, unlike other representatives of the genus, *P.corallicola* is parasitic as an adult. Based on above we assume that *P.corallicola* belongs to another genus. Our conclusion is also supported by the molecular phylogenetic tree (Fig. 10), values of intergeneric p-distances within Leptosomatidae, and interspecies p-distances within *Aborjinia* (Table 3). We propose the new genus *Paraborjinia* gen. nov. for *A.corallicola*.

#### ﻿ Paraborjinia gen. nov.

Taxon classificationAnimaliaEnoplidaLeptosomatidae

3D0816D2-1E6D-5B79-A11F-B9C2D7DB2BF3

https://zoobank.org/5662ABEE-8888-4C29-ACF2-6EDDB2A63F0B

##### Type species.

*Paraborjiniacorallicola* (Westerman, de Moura Neves, Ahmed & Holovachov, 2021). Type locality: Atlantic Ocean, Labrador Shelf (60.6083°N, 61.7428°W), 426 m depth. Type host: *Acanellaarbuscula*.

##### Diagnosis.

Parasitic life style. Distinct body pores along the body. Outer labial and cephalic sensilla papilliform, situated in two circles. Amphideal aperture pore-like. Muscular and uniformly cylindrical pharynx. Intestine not modified into trophosome. Hologonic ovaries in females. Presence of caudal glands.

##### Differential diagnosis.

Within the family Leptosomatidae the new genus differs from all genera except *Aborjinia* by having and endoparasitic lifestyle and hologonic ovaries. *Paraborjinia* gen. nov. differs from *Aborjinia* by the position of cephalic sensitive organs (outer labial and cephalic papilla in two separate circles in *Paraborjinia* vs outer labial and cephalic papilla in one circle in *Aborjinia*). *Paraborjinia* gen. nov. differs from *Aborjinia*, *Ananus*, and *Thalassonema* by the parasitic adult (vs free-living in *Aborjinia*, *Ananus*, and *Thalassonema*). *Paraborjinia* gen. nov. differs from *Ananus* by the presence of rectum and anus. In addition, in all described species of *Aborjinia* and *Ananus* the secretory-excretory system is well developed and consists of two prominent cells while in *Paraborjinia* the secretory-excretory system was not found.

## ﻿Discussion

These results of the phylogenetic analyses are only preliminary due to the low number of sequences available. The different sets of species and genera for constructing the SSU and LSU phylogenetic trees, as well as the small number of sequences relative to the total number of species affect the different topologies. It is premature to make solid conclusions about the relationships of genera within Leptosomatidae based on the available data; however, concatenated 18S and 28S rDNA phylogenetic tree showed relatively high support values (Fig. 10). Recent studies have shown that the genus *Aborjinia* belongs to the family Leptosomatidae based on both molecular and morphological characters (Tchesunov et al. 2022) and our SSU and D2-D3 of LSU phylogenetic trees confirm the previous analyses.

The males of *Aborjiniaprofunda* sp. nov. and female *Aborjinia* sp. (specimen М10) have pronounced morphological differences and p-distances (28S and ITS). Moreover, for all known *Aborjinia* isolates, differences in the nucleotide sequences of LSU and ITS are observed (Table 4). The presence of sexual dimorphism is known for nematodes, including leptosomatids, and the values of p-distances are relatively small. In addition to the commonly used phylogenetic analysis and genetic distances, we used Compensatory Base Changes (CBCs) in Internal Transcriber Spacer 2 (ITS2) for species delimitation. ITS2 is useful locus for calculation of lower-level phylogenetic trees in many eukaryotic lineages (Young and Coleman 2004; Ahvenniemi et al. 2009) to predict the ability to interbreed successfully between putative biological species. Organisms that differ by even one CBC in the conserved ITS2 regions (helices 2 and 3) are unable to interbreed (Coleman 2009). At the same time, changes in single stranded region (hemi-CBC) do not contribute to the appearance of CBCs (Caisová et al. 2011) and lead to failure in sexual reproduction (Coleman 2009). Based on this evidence, when CBCs occur among species, Wolf et al. (2013) developed a generalized ‘CBC species concept’. Double-sided changes (CBC) were not found; therefore, there are no strict reasons for classifying the studied individuals as different species, but resolving the issue of the species status of some individuals requires further research.

Our results indicate a rather wide distribution of representatives of the genus *Aborjinia* in the deep-sea communities of the northwestern Pacific, including depths of more than 8000 m. Molecular and morphological (in particular, the two-celled renette, the presence of a spinerette, minute sensory sensilla, normal muscular pharynx) data support the assignment of *Aborjinia* to Leptosomatidae. Analysis of molecular data confirms the independence of the genera *Aborjinia* and *Paraborjinia* and demonstrates clearly supported differences from *Leptosomatides*. We agree with Tchesunov et al. (2022) that morphological uniformity can lead to misidentification of *Aborjinia* specimens, especially if fixed in ethanol. This may lead to an underestimation of the frequency of occurrence, abundance, and diversity of both representatives of the genus *Aborjinia* and parasitic leptosomatids in general.

## Supplementary Material

XML Treatment for Leptosomatidae

XML Treatment for Aborjinia

XML Treatment for Aborjinia profunda

XML Treatment for Aborjinia

XML Treatment for Paraborjinia

## ﻿Additional information

### Data availability

All of the data that support the findings of this study are available in the main text or Supplementary Information.

## Supplementary materials

Supplementary material 1 Light microscopy, DIC. *Leptosomatides* sp., female Data type jpg Explanation note Light microscopy, DIC. *Leptosomatides* sp., female. **A** – anterior end. **B** – posterior end. **C–E** – head. **F** – vulva region. **G** – tail. Scale bars: 100 µm. https://binary.pensoft.net/file/968829This dataset is made available under the Open Database License (http://opendatacommons.org/licenses/odbl/1.0/). The Open Database License (ODbL) is a license agreement intended to allow users to freely share, modify, and use this Dataset while maintaining this same freedom for others, provided that the original source and author(s) are credited.Julia K. Zograf, Alexander A. Semenchenko, Vladimir V. Mordukhovich

Supplementary material 2 Bayesian 18S rDNA phylogeny of the family Leptosomatidae, using the SYM+I+G model of nucleotide substitution Data type pdf Explanation note *noplustaipingensis* (Enoplidae) and *Phanoderma* sp. (Phanodermatidae) were used as outgroups to root the tree. Bayesian posterior probabilities (PP) are given above tree nodes and bootstrap support values found in the ML analysis are shown below nodes. https://binary.pensoft.net/file/968833This dataset is made available under the Open Database License (http://opendatacommons.org/licenses/odbl/1.0/). The Open Database License (ODbL) is a license agreement intended to allow users to freely share, modify, and use this Dataset while maintaining this same freedom for others, provided that the original source and author(s) are credited.Julia K. Zograf, Alexander A. Semenchenko, Vladimir V. Mordukhovich

Supplementary material 3 Bayesian 28S rDNA phylogeny of the family Leptosomatidae, using the SYM+I+G model of nucleotide substitution Data type pdf Explanation note *noplus* sp. (Enoplidae) and *Phanoderma* sp. (Phanodermatidae) were used as outgroups to root the tree. Specimens obtained in this study are indicated in bold. https://binary.pensoft.net/file/968834This dataset is made available under the Open Database License (http://opendatacommons.org/licenses/odbl/1.0/). The Open Database License (ODbL) is a license agreement intended to allow users to freely share, modify, and use this Dataset while maintaining this same freedom for others, provided that the original source and author(s) are credited.Julia K. Zograf, Alexander A. Semenchenko, Vladimir V. Mordukhovich
