# Supplementary figures and images for: ﻿New deep-sea species of Aborjinia (Nematoda, Leptosomatidae) from the North-Western Pacific: an integrative taxonomy and phylogeny
Source: Zookeys. 2024 Jan 17;1189:231–56. doi: 10.3897/zookeys.1189.111825 (PMC10809327; doi:10.3897/zookeys.1189.111825)

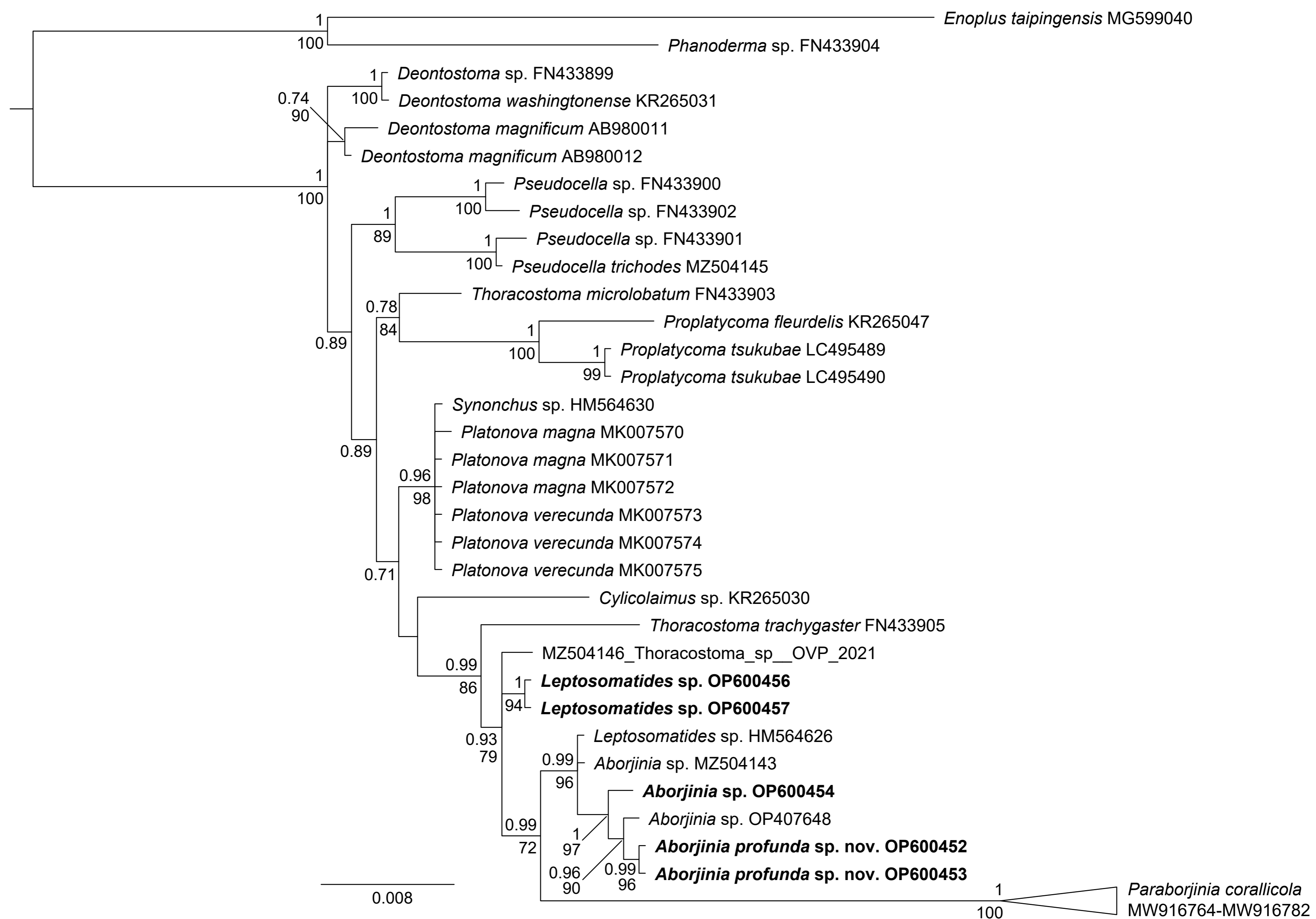

Supplement: Supplementary material 2 — Bayesian 18S rDNA phylogeny of the family Leptosomatidae, using the SYM+I+G model of nucleotide substitution [file zookeys-1189-231_article-111825__-s002.pdf]

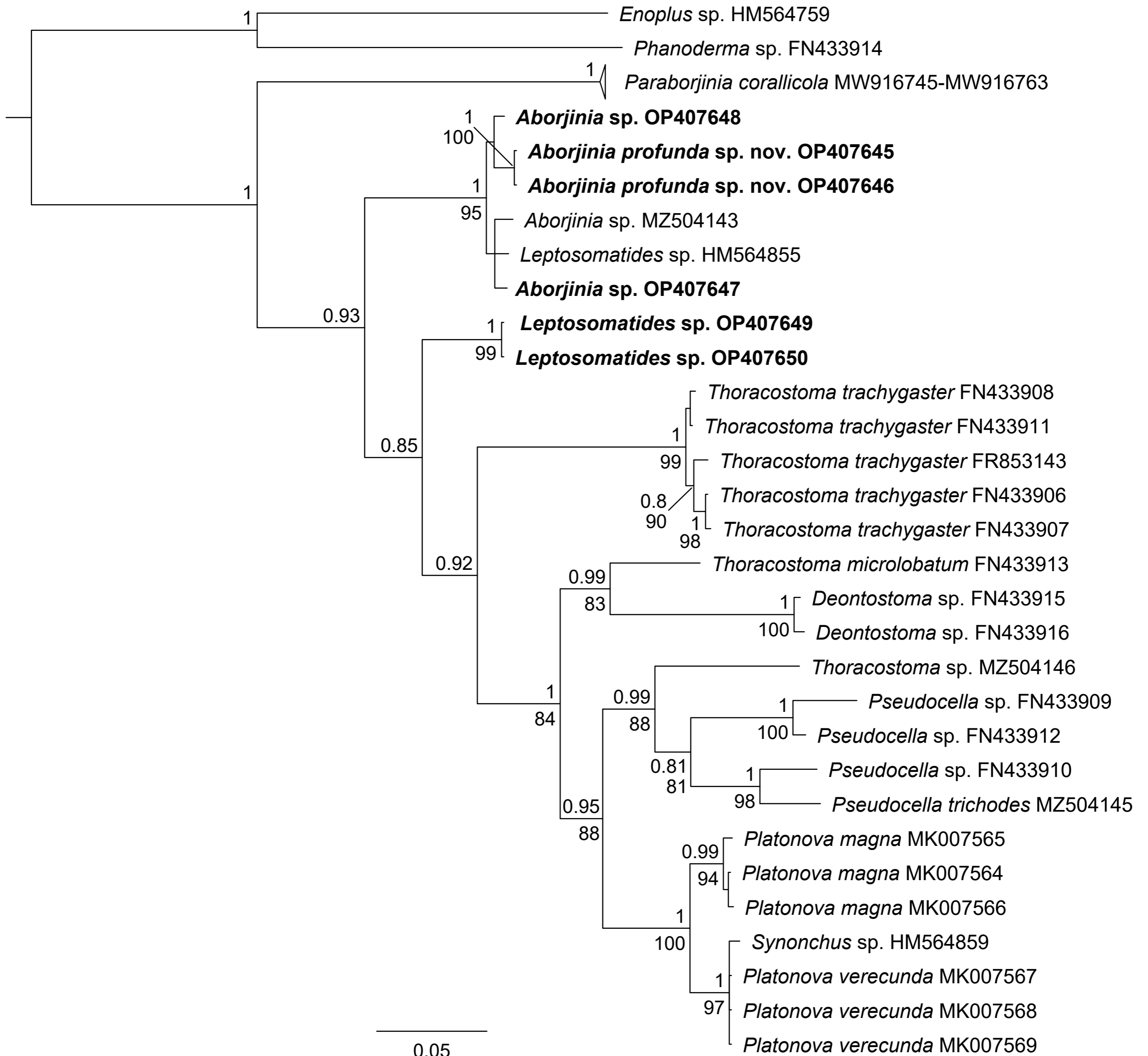

Supplement: Supplementary material 3 — Bayesian 28S rDNA phylogeny of the family Leptosomatidae, using the SYM+I+G model of nucleotide substitution [file zookeys-1189-231_article-111825__-s003.pdf]
